# Wing Defects in *Drosophila xenicid* Mutant Clones Are Caused by C-Terminal Deletion of *Additional Sex Combs* (*Asx*)

**DOI:** 10.1371/journal.pone.0008106

**Published:** 2009-12-01

**Authors:** Kara Bischoff, Anna C. Ballew, Michael A. Simon, Alana M. O'Reilly

**Affiliations:** 1 Department of Biological Sciences, Stanford University, Stanford, California, United States of America; 2 Fox Chase Cancer Center, Philadelphia, Pennsylvania, United States of America; University of Birmingham, United Kingdom

## Abstract

**Background:**

The coordinated action of genes that control patterning, cell fate determination, cell size, and cell adhesion is required for proper wing formation in *Drosophila*. Defects in any of these basic processes can lead to wing aberrations, including blisters. The *xenicid* mutation was originally identified in a screen designed to uncover regulators of adhesion between wing surfaces [1].

**Principal Findings:**

Here, we demonstrate that expression of the βPS integrin or the patterning protein Engrailed are not affected in developing wing imaginal discs in *xenicid* mutants. Instead, expression of the homeotic protein Ultrabithorax (Ubx) is strongly increased in *xenicid* mutant cells.

**Conclusion:**

Our results suggest that upregulation of Ubx transforms cells from a wing blade fate to a haltere fate, and that the presence of haltere cells within the wing blade is the primary defect leading to the adult wing phenotypes observed.

## Introduction

Wing development in *Drosophila* depends on intricate coordination of complex developmental processes, including cell fate determination, growth, and adhesion. *Drosophila* wings develop from imaginal discs, which are ectodermally-derived structures that develop within the larval casing. The blade of the wing is formed when the disc folds on itself and telescopes out, after axes are established and the majority of cell divisions have occurred. The resulting wing is just two epithelial cell layers thick, with adhesion proteins attaching the layers together [2].

Signals that control axis formation, patterning, cell division, and adhesion during wing formation have been identified. In the *Drosophila* wing imaginal disc, three axes are defined by early-expressed signals. These axes provide wing cells with positional information used for cell fate determination. After axial patterns are established, imaginal disc cells proliferate and grow in response to spatially and temporally controlled signals. By the beginning of pupariation, most cell division is complete and the wing disc undergoes dramatic morphological changes. Cell shape changes promote folding of the wing disc along the margin, resulting in the apposition of the dorsal and ventral surfaces of the wing and wing blade formation [2].

Both the changes in morphology and adhesion between wing surfaces depend on interaction between integrin adhesion molecules and the basal extracellular matrix [2], [3], [4], [5]. Defects in integrin-mediated adhesion or downstream signaling molecules lead to defective apposition of the wing surfaces, resulting in the formation of blisters in the wing blade. This striking phenotype has been utilized in genetic screens designed to identify new integrin interacting genes. Two independent screens were conducted in which mutant clones of cells were generated within the developing wing epithelium [1], [6]. Mutations that lead to the formation of blisters within the adult wing were selected for further analysis. Several important integrin pathway components were isolated using this approach, including three integrin subunits (*myospheroid* (βPS), *multiple edematous wings* (*mew*, αPS1), and *inflated* (*if*, αPS2)), as well as downstream regulators of integrin-mediated adhesion including Talin (*rhea*) [7], Tensin (*blistery*) [8], and PINCH (*steamer duck*) [9]. In addition, *blistered*, the *Drosophila* homolog of serum response factor, was shown to regulate expression of integrins and other adhesion components in the developing wing disc [10], [11].

Several genes isolated in wing blister screens have not yet been cloned or characterized. One allele of *xenicid* was isolated (Prout et al. 1997, FBgn0020770) that was found to cause blisters similar to those observed for integrin mutants, suggesting a potential role for *xenicid* in integrin regulation, adhesion, or signaling. Here, we have characterized the wing defects of *xenicid* mutants in detail and identified the gene as *Additional sex combs* (*Asx*, FBgn0000142, CG8787), a suppressor of *Ultrabithorax* (*Ubx*) gene expression in the wing imaginal disc.

## Results

### *xenicid* Is a Loss-of-Function Allele of *Additional Sex Combs*

**Figure 1:**
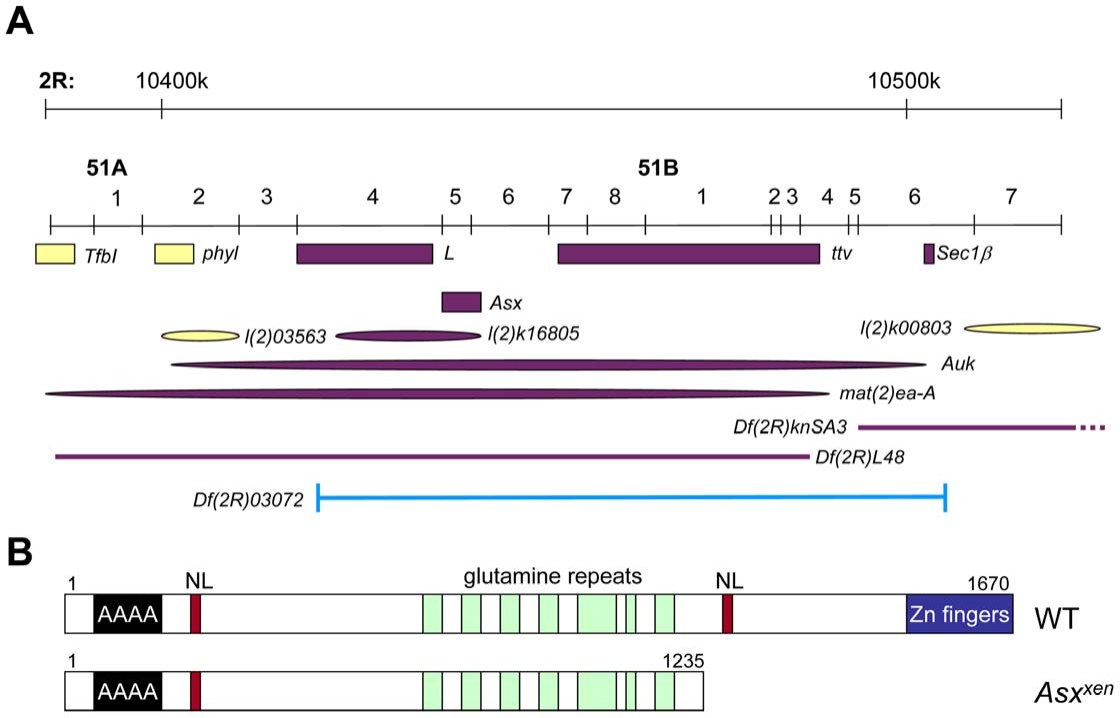
Identification of *xenicid* as an allele of *Additional Sex Combs* (*Asx*). A) Maps of the genomic region from 50F-51B and nucleotides 10,400,000–10,500,000 on the right arm of the second chromosome, adapted from www.flybase.com. Genes (boxes), P-element insertions (ovals) and deficiencies (lines) that fail to complement *Df(2R)03072*/*CyO* are colored purple. P-element insertions or genes that complement are colored yellow. The breakpoints of *Df(2R)03072* are shown in blue. B) Schematic diagram of WT [18] and predicted mutant Asx proteins. Polyalanine (black box), nuclear localization sequences (red boxes), glutamine repeats (green boxes), and a cysteine-rich region predicted to form a double zinc finger (blue box) are indicated. The 14-base-pair deletion in exon 8 of *Asx^xen^* is predicted to result in production of a truncated version of Asx (1235 aa vs. 1670 aa in the wild-type protein) that lacks the C-terminal nuclear localization sequence and Zinc finger domains.

Prout and colleagues mapped the original allele of *xen* (*xen^72−3^*) to 51A-51C on the second chromosome by failure to complement the lethality of *Df(2R)03072* ([1], Figure 1A). *Df(2R)03072* was generated through P-element insertional mutagenesis screens for lethal mutations on the second and third chromosomes [12], [13]. Polytene chromosome analysis revealed a deletion of the 51A-C region. In addition, *Df(2R)03072* retains an inserted P-element. Failure to complement (*xen^72−3^*) might be due either to loss of the deleted region or to disruption of gene function by the presence of the P-element. Therefore, we first mapped the P-element insertion site to the first intron of the *Lobe* gene at position 51A, with the 5′ end of the P-element at nucleotide 10370053 on the right arm of the second chromosome. *Lobe* mutations cause a small-eye phenotype and are not lethal [14], [15], in contrast to the phenotypes observed in *xen* mutants [1]. Since the P-element insertion was not the likely cause of the failure of *Df(2R)03072* to complement *xen^72−3^*, we mapped the breakpoints of the deficiency region using lethal complementation tests between *Df(2R)03072* and genes in the *Lobe* chromosomal region. *Df(2R)03072* fails to complement eight genes in 51A-51C, including *tout velu* (*ttv*), *Additional Sex Combs (Asx),Lobe (L), auk, mat(2)ea-A, sec61b, l(2)k03906*, and *l(2)k16805* (Figure 1A). Lethal mutations in genes located 5′ to Lobe (*TfBl*, *phyllopod* (*phyl*), and *l(2)03563*) complemented *Df(2R)03072*, suggesting that the P-element inserted in Lobe defines the 5′ end of the deficiency. All of the lethal genes we tested that are located 3′ of Lobe failed to complement *Df(2R)03072* except *l(2)k00803*. These data are consistent with deficiency breakpoints at the 3′end of the P-element inserted in Lobe and between *sec61b* (located at base pairs 10506232–10507189 on the second chromosome (release r5.22)) and *l(2)k00803* (mapped to 51B6) (Figure 1A).

Next, we performed lethal complementation tests between *xen^72−3^* and mutants in each of the eight genes that are deleted in *Df(2R)03072*. *xen^72−3^* failed to complement *Df(2R)03072*, consistent with previously reported results [1]. In addition, *xen^72−3^* failed to complement a loss-of-function allele of Asx (*Asx^XF23^*, [16], [17]), suggesting that *xen^72−3^* is an allele of *Asx*. A second new *Asx* allele (*Asx^C849^*) that we isolated in an unrelated screen (O'Reilly, AM and Simon, MA, unpublished) also failed to complement *Df(2R)03072, xen^72−3^*, and *Asx^XF23^*.

*Asx* contains eleven exons that span six kilobases. The predicted protein product is 1670 amino acids including a polyalanine stretch near the N-terminus, seven glutamine repeats, and a thirty-two amino acid cysteine cluster near the C-terminus (Figure 1B). The cysteine cluster is predicted to form a double zinc-finger structure that may be involved in DNA binding. Additionally, there are two nuclear-localization signals, one near each end of the protein (Figure 1B, [18]).

To verify that *xen^72−3^* represents a new allele of *Asx*, the coding exons of *xen^72−3^* were sequenced, revealing a fourteen base-pair deletion (nucleotides 3706–3718 of the coding region) in the eighth exon in *Asx*. The deletion leads to a frame shift and an early stop codon (Figure 1B). The predicted mutant Asx protein lacks the C-terminal 435 amino acids of the wild-type protein, including the second nuclear localization sequence and the highly conserved cysteine cluster, which is thought to mediate binding to DNA [18]. *Asx^C849^* and *Asx^xen^* exhibit phenotypes that are indistinguishable from the previously characterized amorphic allele, *Asx^XF23^* ([16]), supporting the idea that *Asx^xen^* and *Asx^C849^* represent new loss-of-function alleles of *Asx*.

### Wing Defects in *Asx* Mutants

Next, we characterized the wing defects in *Asx* mutants in detail. Four categories of wing blister were defined in the original screen by Prout and colleagues [1]. Type A blisters can be located anywhere on the wing blade and are relatively round, with normal vein patterning. Type B blisters are phenotypically similar to Type A blisters, but only form when mutant clones are generated on the distal half of the wing. Type C blisters lack the sharp boundaries seen in Type A and B blisters, and they are very large, causing wing crumpling. Finally, Type D blisters exhibit defects in vein patterning in addition to the formation of Type A-like blisters.

All of the genes that have been shown to participate in integrin-mediated adhesion were classified initially as Type A or B blisters [1], [6]. Clones of *xen^72−3^* (now *Asx^xen^*) exhibited Type A blisters and a similar lethal phase to that of integrin pathway regulators [1]. To investigate potential roles for *xen* in integrin regulation, we characterized the wing defects of three loss-of-function *Asx* alleles, *Asx^xen^*, *Asx^C849^* and *Asx^XF23^*.

**Figure 2:**
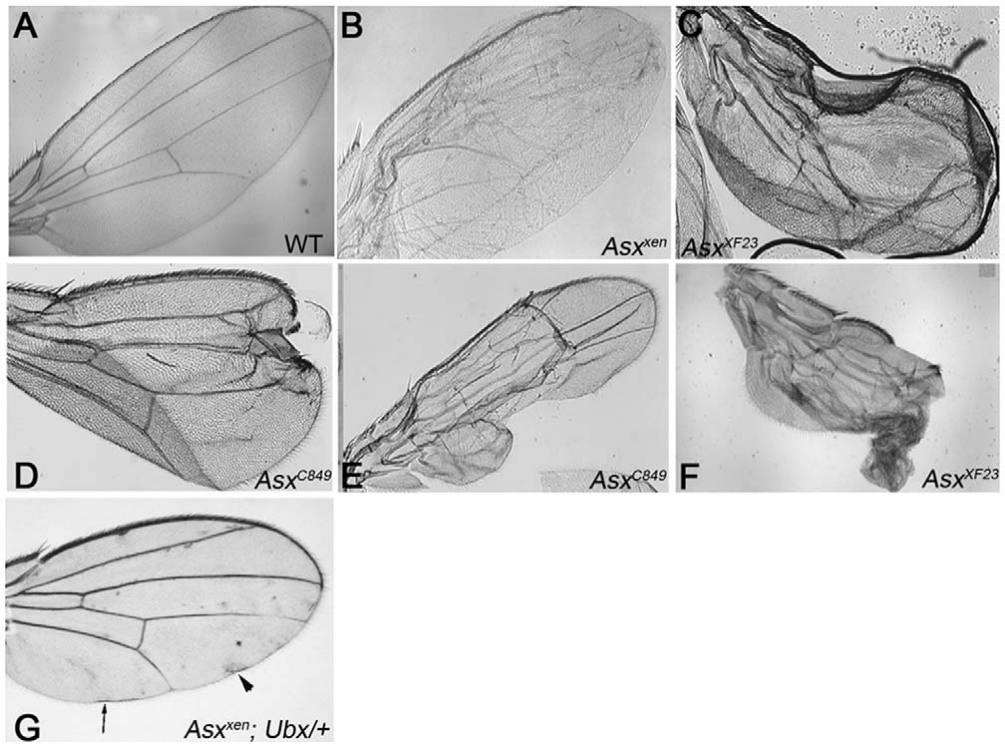
Five phenotypes result from *Asx* mutation in clones of cells in the adult wing blade. A) Wild-type wing with no defects. B) Wing bearing *Asx^xen^* mutant cells with a Type A wing blister (see text). C) Crumpled wing (Type C blister) resulting from *Asx^XF23^* mutation. D) Bent wing bearing *Asx^C849^* mutant clones. E) Dual-lobed wing bearing *Asx^C849^* mutant clones. F) Crumpled wing with necrotic cells resulting from *Asx^XF23^* mutation. All five phenotypes were observed at similar penetrance in the three different *Asx* alleles analyzed. G) “Rippled” Ubx/+ wing bearing *Asx^xen^* mutant cells (genotype is *Flp*; *FRT^42D^ Asx^xen^*/*FRT^42D^ GFP*; *Ubx/+*). Arrowhead points to a ripple associated with a clone of small cells. Arrow indicates a ripple lacking associated small cells.

Although Type A blisters were observed at a low frequency (9.7%) in homozygous *Asx^xen^* mutant clones (Figure 2B, 5C), four additional categories of wing defects were observed. More than one-fifth of the *Asx^xen^* clones generated led to crumpling of the wing (20.6%, Figure 2C, 5C), a characteristic of the original Type C blisters. Wings that were bent or folded along the clone were also observed (13.8%, Figure 2D, 5C). A small number of wings bearing *Asx^xen^* mutant clones exhibited dual-lobes (5.0%, Figure 2E, 5C), a phenotype that is correlated with pattern duplications. Finally, patches of necrotic cells associated with *Asx^xen^* mutant clones were observed, also at a low frequency (2.9%, Figure 2F, 5C). *Asx^C849^* and *Asx^XF23^* mutation led to the same panel of defects (Figure 2) and data not shown. Wings with multiple defects were common, and similar defects were never observed in wild-type wings (Figure 2A).

### *Asx* Does Not Regulate Integrin Expression or Localization

Whereas the formation of Type A wing blisters in *Asx* mutant clones is reminiscent of the defects caused by integrin mutation, crumpled, bent/folded, dual-lobed, or necrotic wings were not observed in integrin mutant clones ([1], [6], [19], [20], [21], [22], [23], and data not shown). Thus, *Asx* may regulate multiple processes during wing development, perhaps including integrin-mediated events. Consistent with this idea, Asx protein binds to many locations on polytene chromosomes isolated from salivary glands, including the X chromosome in the region of 7A, where the gene that encodes βPS (*myospheroid* (*mys*)) is located [18].

**Figure 3:**
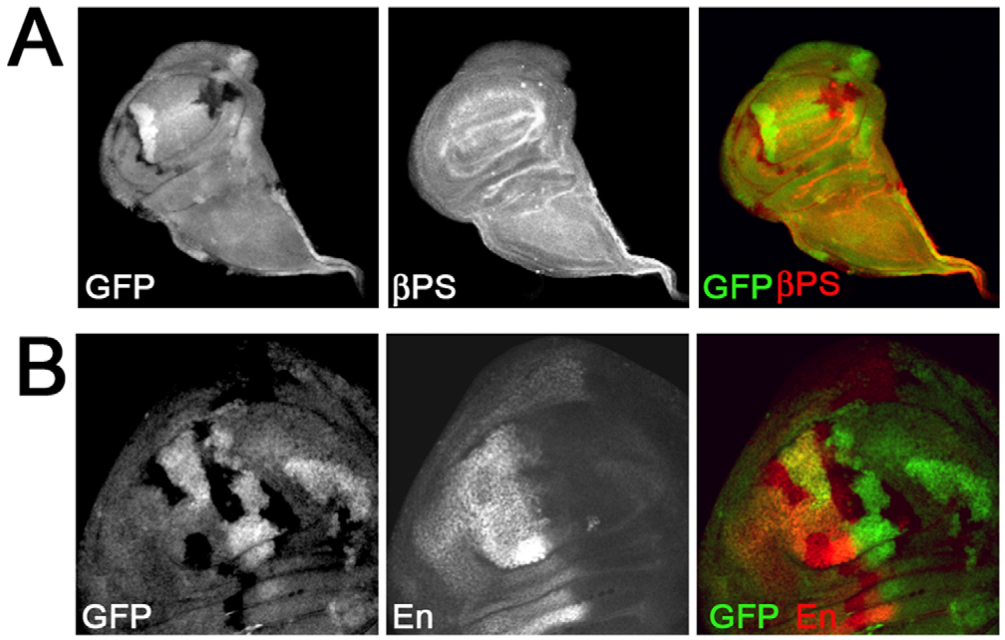
*Asx* mutation has no effect on βPS integrin or Engrailed expression. Wing discs bearing *Asx* mutant clones (lacking GFP, green) were immunostained with anti-βPS (A) or anti-Engrailed (B) antibodies.

To test whether βPS levels or localization were affected by Asx mutation, we immunostained wing discs bearing *Asx* mutant clones with anti-βPS integrin antibodies. No difference in expression level or localization was observed within versus outside the mutant clone (Figure 3A), indicating that *mys* is not an Asx target in the wing imaginal disc. βPS is an obligate subunit of both of the integrins known to mediate adhesion of wing blade cells to the basement membrane (αPS1βPS and αPS2βPS, [20], [21], [22], [23], [24]. If other integrin subunits or ECM ligands were critical Asx targets, we would expect to see changes in βPS levels or localization. Moreover, none of the other integrin subunits or ECM ligands is located near Asx chromosomal binding sites in salivary gland polytene chromosomes [18]. Together, these results suggest that *Asx* mutant defects in the wing are not due to changes in integrin expression or function in the developing wing disc.

### *engrailed* Expression Is Not Affected by *Asx* Mutation

In the wing imaginal disc, three axes are defined by early-expressed signals [25]. These axes provide wing cells with positional information so that cell fates can be determined correctly. The A-P axis is defined by *engrailed* (*en*) expression in the posterior half, or compartment, of the wing. En induces a cascade of events that patterns the entire A-P axis, and ectopic En expression in the anterior compartment of the wing leads to pattern duplications.

*Asx* was originally characterized as a member of the Polycomb group (PcG) of transcriptional repressors [18], [26], [27], [28], [29]. Core PcG genes modify chromatin structure to prevent transcriptional activation of key target genes, including the well-characterized homeotic genes that are critical for developmental patterning and cell fate determination [30]. Increasing evidence has demonstrated the role of PcG genes in controlling *engrailed* expression. For example, mutation of PcG member *polyhomeotic (ph*) in the anterior compartment of the wing results in ectopic expression of *en*, which leads to formation of multiple A-P boundaries and therefore pattern duplication [31].

We found that *Asx* mutation led to the formation of dual-lobed wings at a low frequency (Figure 2E), suggesting that a pattern duplication may have occurred. Moreover, an Asx binding site exists just upstream of *en* according to a polytene chromosome map [18]. Therefore, we tested whether Asx regulates expression of *en* in the developing wing disc. Surprisingly, we saw no effect on En expression levels or localization in *Asx* mutant clones (Figure 3B). Asx-dependent changes in En expression might occur at a low frequency or at later stages of development, leading to the small percentage of dual-lobed wings observed. However, the observation that En patterns are predominantly normal in Asx mutant imaginal disc cells suggests that other mechanisms are responsible for the majority of adult wing phenotypes observed.

### *Asx* Mutant Cells Are Smaller than Their Neighbors

**Figure 4:**
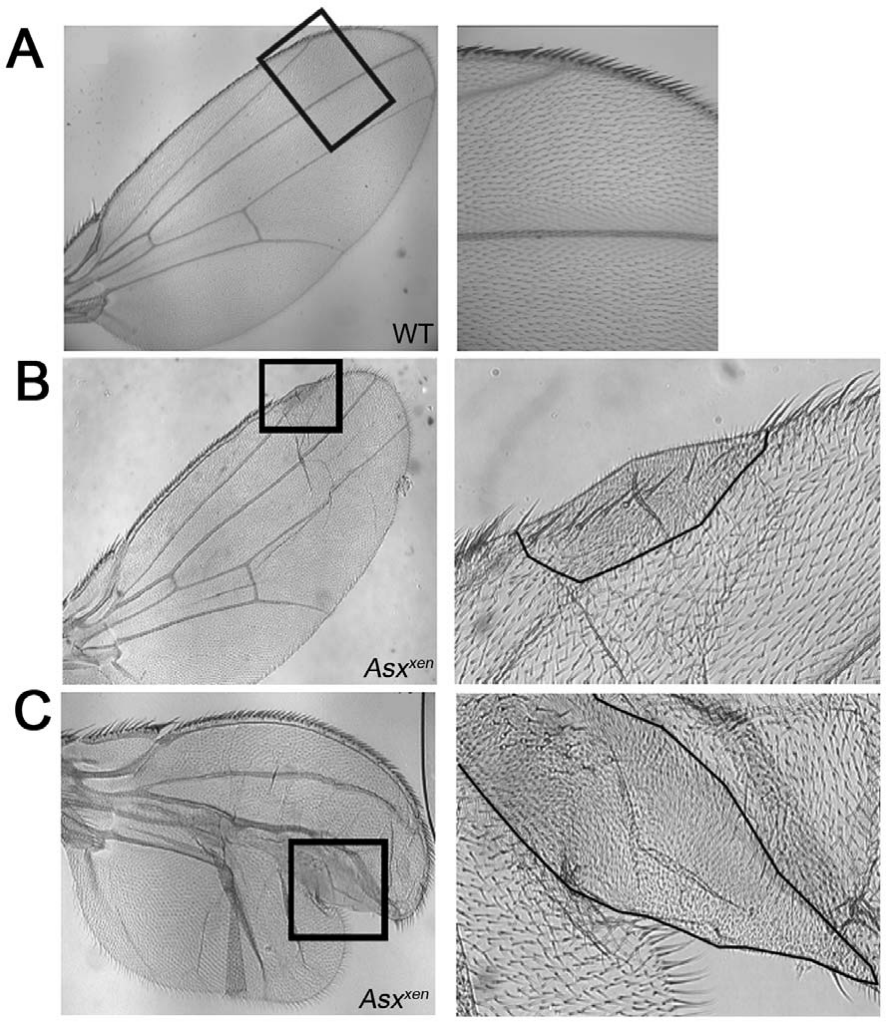
*Asx* mutant clone adult wings have patches of small cells containing more than one hair. Boxed areas in wing blades are magnified at the right. Small cell clones are outlined in black. A) Wild type. B,C) Wings bearing *Asx^xen^* mutant clones with small cells. Clone on edge of wing (B) and dual-lobed wing with small cells in the cleft between the lobes.

**Figure 5:**
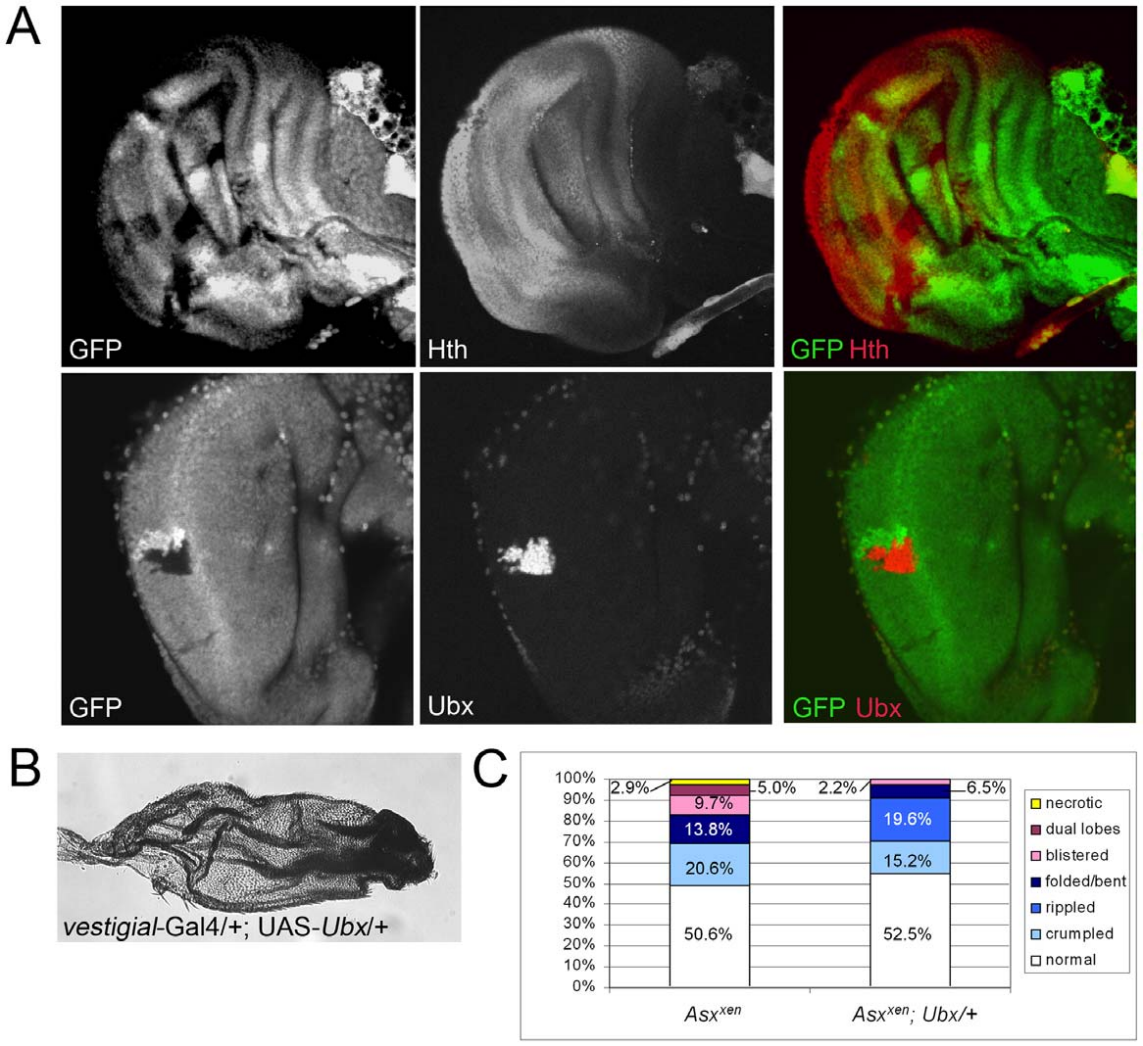
The primary defect in *Asx* mutants is upregulation of *Ubx*. A) Larval wing discs bearing *Asx^xen^* mutant clones (lacking GFP, green) were immunostained stained with anti-Hth (top) or anti-Ubx (bottom). Hth is expressed in the presumptive wing hinge in wild-type larval wing discs, and no effect is seen on Hth expression in *Asx* mutant clones. Ubx is expressed ubiquitously in haltere cells in imaginal discs, but is not normally expressed in wild-type wing blade cells. Ubx expression is strongly up-regulated within *Asx* mutant clones. B) Expression of UAS-*Ubx* throughout the wing blade using *vestigial*-Gal4 leads to wing defects that are indistinguishable from those seen in *Asx* mutant clones. C) Quantitation of wing phenotypes associated with *Asx^xen^* mutant clones in flies that are wild-type at the *Ubx* locus (column 1, N = 383) or heterozygous (column 2, N = 46). Phenotypes are shown in order of increasing severity from bottom to top. Reduction of *Ubx* levels partially suppresses *Asx^xen^* defects.

To further investigate the cellular defects in *Asx* mutant wing clones, we examined adult wings at high magnification (Figure 4). Nearly all of the phenotypically abnormal wings had patches where wing hairs were significantly smaller than average and close together, as if the cells in those regions were small (Figure 4B). In dual-lobed wings, patches of small cells were found at the furrow between the two lobes (Figure 4C), forming a cleft that split the normal wing blade. In other wings, normal sized cells surrounded an area where the cells were small, causing crumpling of the wing. Thus, the presence of clones of small cells within the plane of the normal wing blade may be a primary defect that contributes to the observed phenotypes. No differences in cell size were observed in *Asx* mutant clones in larval or pupal wing discs (Figures 3,5), and data not shown, and the number of cells within mutant clones was comparable to wild-type at these stages. Thus, the cell size defects observed in adult wings must arise due to defects in late-stage growth of wing cells.

### Asx Suppresses Ubx Expression in Wing Cells, Preventing Adoption of a Haltere Fate

An obvious explanation for the small size of the cells within *Asx* mutant clones is that wing blade cells assume the fate of smaller cells, leading to mismatches during wing surface apposition or to developmental differences that cause the observed defects. Cell fate transformation is a hallmark of mutation in PcG and TrxG genes, and is how the genes in these groups were originally identified. To examine this possibility, we tested whether the cells within *Asx* mutant clones had assumed two possible alternative fates, wing hinge fate or haltere fate, both of which render cells that are smaller than wing cells. *Homothorax* (*Hth*) is used as a marker of wing hinge fate [32], [33], as it is expressed ubiquitously there. *Asx* mutant clones were immunostained with antibodies against Hth, which revealed that Hth expression levels were not affected in *Asx* mutant clones (Figure 5A). In contrast, dramatic upregulation of the haltere fate marker, Ultrabithorax (Ubx), was observed in *Asx* mutant clones (Figure 5A), consistent with a previous report [34]. Ectopic expression of *Ubx* throughout the wing blade in a *vestigial* pattern lead to similar defects (Figure 5B), including small, crumpled, wings with small necrotic cells. These results support the model that ectopic expression of *Ubx* in *Asx* homozygous mutant clones is the primary defect leading to wing blade abnormalities [34].

To test this model directly, we generated Asx mutant wing clones in flies with reduced Ubx function. Wing defects still were observed when *Asx^xen^* mutant clones were generated in flies lacking one copy of Ubx (Flp; FRT^42D^ *Asx^xen^*./FRT^42D^ GFP; Ubx/TM6b). However, the severity of the phenotypes was reduced such that no necrotic or dual-lobed wings were observed (Fig. 5C). The percentage of wing blisters or folded/bent wings was reduced by 4.4 and 2.1 fold, respectively. Fewer flies had crumpled wings (20.6% vs. 15.2% in Ubx heterozygotes), but 19.6% of flies had “rippled” wings that likely represent very mild crumpling (Fig. 2G, 5C). In some cases, ripples were associated with visible clones containing small cells (Fig. 2G), arrowhead whereas other ripples were not associated with the presence of smaller cells (Fig. 2G), arrow. This suggests that a wing to haltere transition caused by *Asx^xen^* mutation was suppressed by reducing the level of Ubx expression. We attempted to generate Asx mutant clones in flies bearing viable, homozygous Ubx mutations (*Flp*; *FRT^42D^ Asx^xen^*./*FRT^42D^ GFP*; *Ubx^bx3^ Ubx^pbx1^*/*Ubx^101^*) but no viable adults were recovered (N = 175). Necrotic third instar homozygous *Ubx* mutant larvae that lacked expression of *Tubby*, a marker for the TM6b balancer, were observed, suggesting that loss of one copy of *Asx* in the *Ubx* mutant background caused lethality at this stage of development. Most likely, Asx and Ubx both are required during larval stages and reducing the function of both genes prevents developmental progression.

## Discussion

Wing blister screens have been strikingly successful tools for identifying new genes involved in important developmental processes such as cell fate determination, growth, and adhesion. Components of two different adhesion mechanisms have been identified in these screens, integrin-mediated basal adhesion (*myospheroid* (βPS), *multiple edematous wings* (*mew*, αPS1), *inflated* (*if*, αPS2), Talin (*rhea*) [7], Tensin (*blistery*) [8], PINCH (*steamer duck*) [9], and *blistered* [10], [11]), and apical adhesion to the cuticle (*papillote*, *piopio*, and *dumpy* [35]). The discovery and functional analysis of these genes has provided important information regarding the role of dynamic changes in adhesion during wing development. Additional genes involved in Notch signaling (*Delta*, *mastermind* [1]), RNA processing (*held out wings*/*scorpion* [36]), and a gene that may participate in cell cycle regulation (*moa/sac*)[37] also have been identified. Thus, insight into multiple developmental processes that are required for wing formation has been gained through the identification of genes isolated in these screens.

Here, we have characterized the defects associated with *xenicid* mutation, a gene that was reported to cause Type A blisters (round, normal vein patterning) in the original wing blister screen [1]. Whereas some wings bearing *xen* mutant clones exhibited Type A wing blisters, phenotypes that included bending, dual-lobes, crumpling, and necrosis also were observed (Figure 2, 5C). These defects are associated with upregulation of Ubx (Figure 5B), which normally is required for haltere development but is suppressed in developing wing blade cells. Loss of Ubx expression in the developing haltere causes transformation of haltere cells into wing blade cells, resulting in the formation of two sets of wings [38], [39], [40], [41]. Conversely, overexpression of Ubx in the wing promotes adoption of a haltere fate [42], [43], [44], [45], [46], [47], [48]. Thus, upregulation of Ubx in wing blade cells lacking *xen* likely causes a wing blade to haltere transformation that is responsible for the array of defects observed.

Although wing and haltere precursor cells in third instar larvae are morphologically indistinguishable, the expression of Ubx in late third instar and early pupal stages controls vastly different developmental events throughout pupal stages [49]. Starting in the third instar larval stage, developing wing blade cells lack expression of Ubx and proceed through several morphological changes. During pupal stages, hexagonal cells, each with a single hair, grow and change shape, forming star-like cells that interdigitate with their neighbors. The dorsal and ventral surfaces of the wing blade are closely apposed during early pupal stages, separate briefly, and then become tightly attached through integrin mediated interactions. Wing blade cells continue to grow until eclosion, resulting in large cells with a single hair projecting from the apical surface [2], [23], [49].

The development of Ubx-expressing haltere cells is markedly different. Haltere cells grow very little during pupal stages, instead remaining similar in size to their larval precursors. Whereas wing cells develop through hexagonal and star-shaped morphologies, haltere cells are cuboidal. Moreover, the dorsal and ventral haltere surfaces remain separate throughout development, with integrins mediating attachment to the central haltere matrix rather than dorsal cells. Consistent with these morphological differences, aberrant upregulation of Ubx in the developing wing blade prevents adhesion between wing surfaces, leaving a hollow cavity [49].

Together, these developmental differences explain the array of phenotypes we observed in *xen* mutant cells. No defects in cell size or morphology were observed in *Asx^xen^* mutant cells in larval wing discs or pupal wings where wing cells and haltere cells are expected to be similar [49]. The blisters, crumpling, dual lobes, and bending/folding phenotypes associated *Asx^xen^* mutation therefore must have developed during pupal stages. The presence of small, cuboidal *Asx^xen^* mutant cells on one surface of the wing blade likely prevents proper apposition of larger, star-shaped wing blade cells on the opposite wing surface. Moreover, the presence of haltere cells likely prevents the attachment, detachment, and adhesion steps that occur during wing formation, instead maintaining an open cavity between surfaces. The inability of smaller and morphologically distinct haltere cells to match wing cells in the same or opposite planes also explains other defects observed in *xen* mutant clones, including crumpling, bending, and dents or dual lobes in the wing surface. Consistent with this model, the presence of non-uniformly sized cells in particular wing compartments leads to crumpling, bending, and other distortions [50], [51]. Previous reports that high levels of Ubx expression lead to cell death provide an explanation for the necrotic cells observed in a small percentage of *xen* mutant clones [49]. Together, these observations suggest that all of the defects observed in *xen* mutant clones are due to upregulation of Ubx and an accompanying wing blade to haltere transformation.

*xenicid* is a newly identified loss-of-function allele of *Additional Sex Combs* (*Asx*), a gene originally identified as a member of the Polycomb Group (PcG) of homeotic regulators. *Asx* is a unique member of the PcG complex in that it does not always act in conjunction with the other PcG members, but also participates with the trithorax Group (trxG) [18], [29], [30], [52]. Complexes of PcG and trxG proteins have been identified that bind directly to chromatin through PcG and trxG response elements, respectively. However, Asx has not been shown to be a member of any of the identified core complexes [30], leading to the suggestion that it acts as a cofactor or enhancer of trithorax and Polycomb via mechanisms that are not currently clear. Recently, it was shown that binding of Asx to a well-characterized 25 kb sequence in the *Ubx* promoter region depends on *trithorax* (*trx*) [52], suggesting that Asx cooperates with trxG genes rather than PcG genes for *Ubx* regulation.

Our results support recent studies demonstrating that Asx suppresses *Ubx* expression in the developing wing imaginal disc [34]. *Ubx* suppression likely is the primary role for Asx in the wing disc since overexpression of *Ubx* throughout the wing blade led to phenotypes that were indistinguishable from *Asx* loss-of-function and reduction of Ubx copy number partially suppressed the effects of *Asx^xen^* mutation (Figure 5). Further evidence for this model comes from observations that mutation of multiple PcG genes leads to aberrant Ubx expression in wing imaginal discs. The level of Ubx upregulation correlates with the severity of the adult wing phenotypes observed [34]. Upregulation of Ubx in *Asx* mutant clones is consistent with a genetic role for *Asx* in PcG-mediated suppression of homeotic gene transcription rather than trxG-mediated activation. However, the exact role of Asx in homeotic gene regulation is not clear. Asx might suppress the ability of trxG proteins to promote transcriptional activation of *Ubx* rather than enhance the function of PcG complexes in suppression. Alternatively, Asx may associate with both PcG and trxG proteins, but interactions with PcG proteins occur on *Ubx* promoter elements outside of the 25 kb region analyzed [52]. Further analysis is required to distinguish between these possibilities.

Asx may function by binding to DNA since loss of the predicted zinc finger domains in *Asx^xen^* mutant cells has dramatic effects on Ubx expression and cell fate determination. However, other explanations are possible. Zinc finger domains also can mediate protein-protein interactions, raising the possibility that Asx C-terminal deletion may disrupt an important transcriptional regulatory complex. The C-terminal domain also contains one of two nuclear localization sequences in Asx, suggesting that a truncated protein might fail to enter the nucleus, thus preventing normal transcriptional complexes from forming. Alternatively, deletion of 435 amino acids might destabilize the protein, leading to reduced or absent Asx levels within mutant clones. In any case, loss of the C-terminal region of Asx leads to a loss-of-function phenotype, demonstrating that this domain is essential for Asx function or stability.

Whereas *Ubx* is a well-characterized target of Asx, PcG, and trxG regulation, the significance of association of these proteins with other target sites on polytene chromosomes remains largely unclear. We found no change in expression of three candidate Asx targets, *myospheroid*, *homothorax*, and *engrailed* in *Asx* mutant clones in wing imaginal discs. The lack of *engrailed* regulation by Asx was surprising for two reasons. First, *en* is a previously identified PcG target [53], [54], [55], [56]. Second, previous work demonstrated that reduction of *Asx* levels in embryos bearing weak mutations in *Posterior sex combs* (*Psc*), a member of the PRC1 PcG chromatin binding complex, and *Polycomblike* (*Pcl*), enhanced *en* expression [53]. These observations suggested that Asx might be an important regulator of *engrailed* expression during wing development [34]. Instead, our results are most consistent with a model in which Asx-dependent suppression of *Ubx* in developing wing imaginal discs is responsible for the phenotypes observed. It remains possible that Asx regulates expression of *myospheroid*, *homothorax*, *engrailed* or other genes during pupal or adult stages. Alternatively, direct suppression of *Ubx* and prevention of Ubx-dependent transcriptional and developmental programs within the wing may be the only critical Asx-mediated event during wing development.

## Materials and Methods

### *Drosophila* stocks

The following fly stocks were obtained from the Bloomington Stock Center and maintained under standard culture conditions: *w^1118^, Asx^XF23^*, *Asx^xen^*, *Df(2)03072, L^r^, L^5^, L^02637^, auk^2R-4^, mat(2)ea-A^1^, Sec1β^07214^, yw; l(2)k03906, yw; l(2)k16805, w; P{Gal4-vg-M}, w;FRT^42D^, w hsFlp;FRT^42D^ GFP, w;P[UAS-Ubx], Ubx^bx3^Ubx^pbx1^, and Ubx^101^Ubx^101^*. The *Asx^C849^* allele was generated through ethyl-methyl sulfonate mutagenesis (O'Reilly AM, and Simon, MA, unpublished data). *w;FRT^42D^ Asx/w hsFlp;FRT^42D^ GFP* larvae were heat shocked for 2 hours at 37°C to generate GFP-marked clones.

### Cloning of *xenicid* Allele

The P-element insertion site for *Df(2R)03072* was identified by isolating genomic DNA from *Df(2R)03072*/*CyO* heterozygous flies. Plasmid rescue was performed, and the resulting DNA was sequenced using primers specific to the 5′ end of the P-element. The P-element remaining in *Df(2R)03072* is inserted in the first intron of the Lobe gene, at nucleotide 10370053 on 2R (release r5.22).

Lethal complementation tests were performed to uncover the deficiency associated with *Df(2R)03072* by crossing *Df(2R)03072*/*CyO* females with males of the following genotypes: *L^r^, L^5^, L^02637^/CyO, auk^2R-4^/CyO, mat(2)ea-A^1^/CyO, Sec1β^07214^/CyO, l(2)k03906/CyO, l(2)k16805/CyO, l(2)k00803/CyO, l(2)03563/CyO, phyl^2242^/CyO, Tfb1^06949^/CyO, Asx^XF23^/CyO, Asx^xen^/CyO, Asx^C849^/CyO, Df(2R)knSA3/CyO*, and *Df(2R)L48/CyO*. The recovery of CyO only progeny indicated failure to complement, and indicated the absence of a given gene within the deficiency.

To identify the nature of the mutation in *Asx^xen^/CyO* flies, genomic DNA was isolated using standard protocols. *Asx* exons were amplified using genomic PCR. Exon 8 was amplified with the following primers: 5′-GTCGTCCAATTGGCTCAGCATTCG-3′ and 5′-CGGTCCAATAACCTAGAATACCAGAAC-3′. Sequencing of all the amplified exons revealed a 14 base pair deletion in exon 8, at nucleotides 3706–3718 of the coding region.

### Wing Analysis

Adult wings were extracted in 70% ethanol or isopropanol, mounted onto slides in eupharol or Hoyer's medium, and imaged with a Spot camera attached to a Zeiss Axioplan2 microscope.

For immunostaining of wing imaginal discs, third-instar larvae were dissected in PBS and fixed in 4% formaldehyde in phosphate buffered saline (PBS) for 10 minutes, then washed three times in PBS plus 0.3% Triton X-100 (PBST). After fixation and immunochemical staining, the wing discs were removed and mounted in Vectashield. Wing discs were analyzed using a MRC 1024 confocal laser microscope (BioRad) using CoMOS software.

### Immunochemistry and Imaging

Polyclonal anti-GFP (1∶1000, Invitrogen), anti-Hth (1∶300 dilution, Pai et al., 1998), and monoclonal anti-βPS (1∶1000, gift from Danny Brower[57]), anti-En (1∶100, University of Iowa Developmental Studies Hyridoma Bank (DSHB)), and anti-Ubx (1∶20, [58]), primary antibodies were incubated with fixed tissue for 2 hours at 22°C or overnight at 4°C and then washed three times in PBST. Secondary antibodies (anti-mouse Cy3 and anti-rabbit FITC, 1∶200, Jackson Laboratories) were incubated with primary antibody-labeled tissue for 2 hours at 22°C and then washed three times in PBST before mounting.
